# Supplementary material for: Catalase activity deficiency sensitizes multidrug-resistant Mycobacterium tuberculosis to the ATP synthase inhibitor bedaquiline
Source: Nat Commun. 2024 Nov 13;15:9792. doi: 10.1038/s41467-024-53933-8 (PMC11561320; doi:10.1038/s41467-024-53933-8)
Supplement: Supplementary file 1 — Supplementary Information [file 41467_2024_53933_MOESM1_ESM.pdf]

Catalase activity deficiency sensitizes multidrug-resistant *Mycobacterium tuberculosis* to the ATP synthase inhibitor bedaquiline

Ofori-Anyinam B, Hamblin M, Coldren ML, Li B, Mereddy G, Shaikh M, Shah A, Grady C, Ranu N, Lu S, Blainey PC, Ma S, Collins JJ, Yang JH

SUPPLEMENTARY TABLES

***M. tuberculosis* Strain**	**Genotype**	**Resistance**	**Reference (PMID)**	**Source**
H37Rv	Wild-type H37Rv	---	ATCC #27294	Deborah Hung (Broad Institute)<br>William Jacobs (Einstein)
H37Rv Δ*katG*	H37Rv Δ*katG*	INH^R	32631825	William Jacobs (Einstein)
H37Rv ΔkatG pEmpty	H37Rv Δ*katG* pMSG430	INH^R	---	Christina Stallings (WUSTL)
H37Rv Δ*katG*	H37Rv ΔkatG pMSG430-*katG*	---	---	Christina Stallings (WUSTL)
H37Rv p*Empty*	H37Rv p*EXCF*	---	23823726, 25380655	David Sherman (U Washington)
H37Rv p*furA*	H37Rv p*EXCF-Rv1909c*	INH^R	23823726, 25380655	David Sherman (U Washington)
$mc^2$7902	H37Rv Δ*panCD* Δ*leuCD* Δ*argB*	---	29844114	William Jacobs (Einstein)
$mc^2$8245	$mc^2$7902 Δ2116169-2162530	INH^R	29844114	William Jacobs (Einstein)
H37Rv p*Rv3160c*	H37Rv p*EXCF-Rv3160c*	---	23823726, 25380655	David Sherman (U Washington)
H37Rv p*kmtR*	wild-type katG, inhA, rpoB	---	23823726, 25380655	David Sherman (U Washington)
H37Rv p*prpR*	H37Rv p*EXCF-Rv1129c*	---	23823726, 25380655	David Sherman (U Washington)
TDR-TB-0019	KatG S315T, RpoB L533P	INH^R	22236841	David Alland (Rutgers)
TDR-TB-0077	wild-type *katG, inhA, rpoB*	---	22236841	David Alland (Rutgers)
TDR-TB-0081	wild-type *katG, inhA, rpoB*	---	22236841	David Alland (Rutgers)
TDR-TB-0091	wild-type *katG, inhA, rpoB*	---	22236841	David Alland (Rutgers)
TDR-TB-0126	wild-type *katG, inhA, rpoB*	---	22236841	David Alland (Rutgers)
TDR-TB-0163	wild-type *katG, inhA, rpoB*	---	22236841	David Alland (Rutgers)
TDR-TB-0031	KatG S315T, RpoB S531W	INH^R	22236841	David Alland (Rutgers)
TDR-TB-0042	KatG S315T	INH^R	22236841	David Alland (Rutgers)
TDR-TB-0193	*inhA* C -15 T, RpoB S531L	INH^R	22236841	David Alland (Rutgers)
TDR-TB-0198	KatG S315T, RpoB D516V	INH^R	22236841	David Alland (Rutgers)

Supplementary Table 1. List of strains used in this study.

Strain	INH MIC	RIF MIC	*katG* nucleotide variant	*katG* *amino acid variant*	*inhA* nucleotide variant	*rpoB* nucleotide variant	*rpoB* amino acid variant	Lineage	Geographical Origin
INH-Susceptible									
TDR-TB-0077	0.2	30	wild type	wild type	wild type	wild type	wild type	2/Beijing	South Korea
TDR-TB-0081	0.2	≤10	wild type	wild type	wild type	wild type	wild type	2/Beijing	South Korea
TDR-TB-0126	0.2	≤10	wild type	wild type	wild type	wild type	wild type	4/LAM	Brazil
TDR-TB-0163	≤ 0.05	≤10	wild type	wild type	wild type	wild type	wild type	4/Haarlem	Peru
INH-Resistant									
TDR-TB-0019	3.2	120	AGC 315 ACC	Ser 315 Thr	wild type	CTG 533 CCG	Leu 533 Pro		Azerbaijan
TDR-TB-0031	> 3.2	> 120	AGC 315 ACC	Ser 315 Thr	wild type	TCG 531 TGG	Ser 531 Trp		Kazakhstan
TDR-TB-0042	> 3.2	≤ 10	AGC 315 ACC	Ser 315 Thr	wild type	wild type	wild type	1/East African Indian	Bangladesh
TDR-TB-0193	> 3.2	> 120	wild type	wild type	C -15 T	TCG 531 TTG	Ser 531 Leu	4/LAM	Portugal
TDR-TB-0198	3.2	> 120	AGC 315 ACC	Ser 315 Thr	wild type	GAC 516 GTC	Asp 516 Val	4/LAM	Peru

Supplementary Table 2. Information on isoniazid-resistant and isoniazid-susceptible TDR-TB strains used in this study.

SUPPLEMENTARY FIGURES

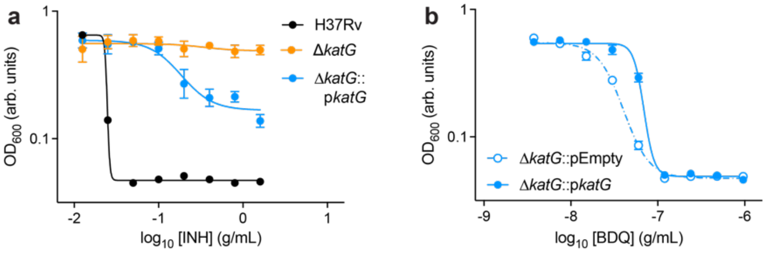

Supplementary Fig. 1. Catalase activity deficiency sensitizes drug-resistant *Mycobacterium tuberculosis* to bedaquiline. a, Δ*katG* cells are resistant to INH relative to wild-type cells in 8-day growth inhibition dose-response experiments. *katG* complementation by transformation with pMSG430-*katG* partially restores INH susceptibility in Δ*katG* cells. **b**, *katG* complementation in Δ*katG* cells decreases BDQ sensitivity relative to empty vector Δ*katG* control cells in 8-day growth inhibition dose-response experiments. n = 3 biological replicates for all experiments. Data depicted as mean ± SEM. Source data are provided in the Source Data file.

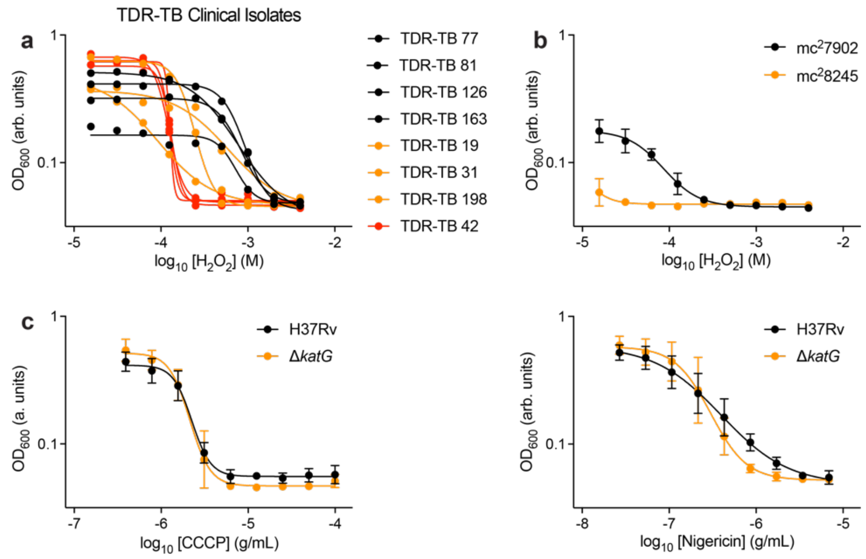

Supplementary Fig. 2. Catalase activity-deficient cells are sensitized to oxidative stress. a, 8-day H_2O_2 growth inhibition dose-response experiments for individual TDR-TB clinical strains (Fig. 2b). MDR (orange) and non-MDR INH-resistant (red) clinical strains are hypersensitive to H_2O_2 relative to INH-susceptible (black) clinical strains. **b**, KatG-deficient mc^{2}8245 cells are hypersensitive to H_2O_2 relative to KatG-replete mc^{2}7902 cells in 8-day growth inhibition dose-response experiments. **c**, Δ*katG* cells are not sensitized to carbonyl cyanide m-chlorophenyl hydrazone (CCCP) (left) or to nigericin (right) relative to wild-type cells in 8-day growth inhibition dose-response experiments. n = 4 biological replicates for non-MDR INH-resistant TDR-TB 42 (red). n = 1 biological replicate for all other TDR-TB clinical strains. n = 3 biological replicates for experiments involving wild-type or Δ*katG* H37Rv cells. Data depicted as mean ± SEM. Source data are provided in the Source Data file.

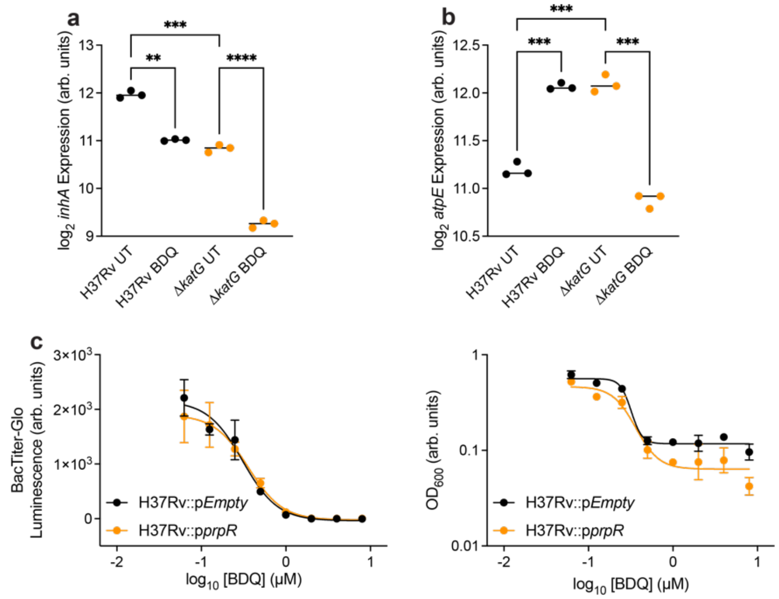

Supplementary Fig. 3. Transcriptional programs induced by catalase activity deficiency sensitize Mtb to bedaquiline. a, BDQ treatment decreases *inhA* expression in wild-type and Δ*katG* cells as measured by RNA sequencing. **b**, BDQ treatment decreases *atpE* expression in Δ*katG* cells as measured by RNA sequencing. Expression data reported as smooth quantile normalized $\log_2$ sequencing counts. **c**, *prpR* over-expression does not sensitize cells to BDQ-inhibited ATP synthesis or growth in 7-day dose-response experiments as determined by BacTiter-Glo and optical density. Brown-Forsythe and Welch ANOVA tests were performed on RNA expression data with comparisons between BDQ-treated and untreated cells wild-type and Δ*katG* cells or between untreated wild-type and Δ*katG* with Dunnett's T3 multiple comparisons test FDR correction, as indicated. n = 3 biological replicates for all experiments. **: $p \leq 0.01$, ***: $p \leq 0.001$, ****: $p \leq 0.0001$. Data depicted as mean ± SEM. Source data are provided in the Source Data file.

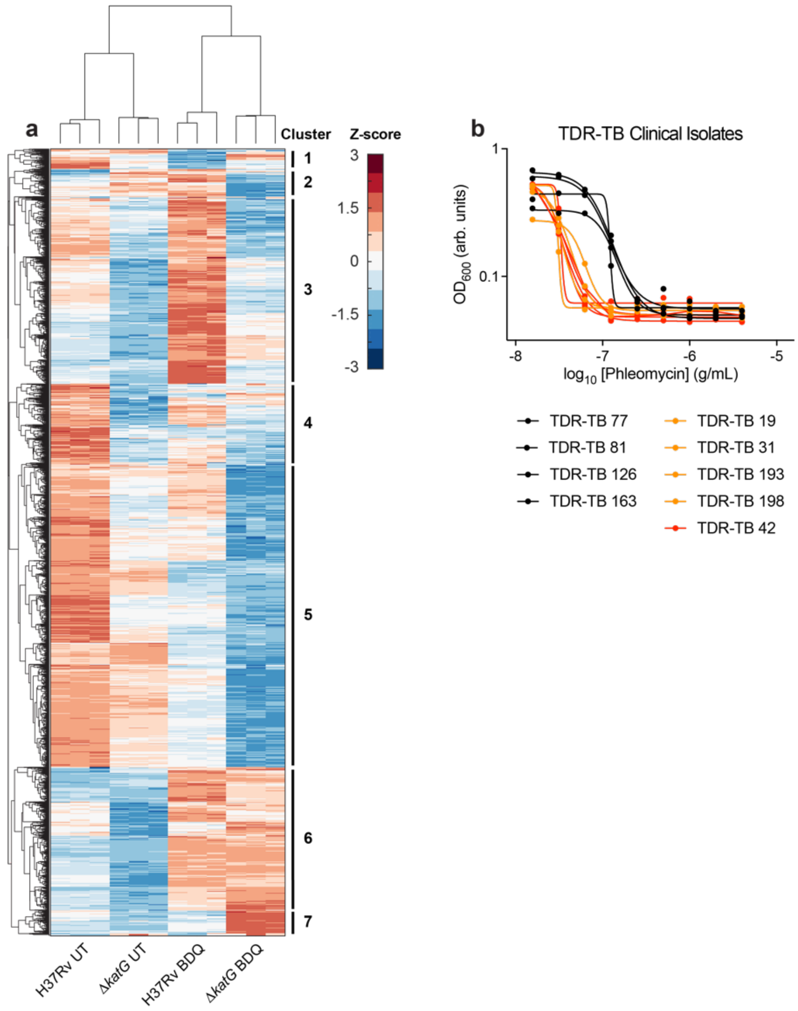

Supplementary Fig. 4. Catalase activity deficiency sensitizes Mtb to DNA damage. a, Smooth quantile normalized RNA sequencing expression data from BDQ-treated and untreated wild-type and Δ*katG* H37Rv cells. Clusters defined by hierarchical clustering, illustrating differences in BDQ-induced expression changes between H37Rv and Δ*katG* cells. n = 3 biological replicates. **b**, 8-day phleomycin growth inhibition dose-response experiments for individual TDR-TB clinical strains (Fig. 4d). MDR (orange) and non-MDR INH-resistant (red) clinical strains are hypersensitive to phleomycin relative to INH-susceptible (black) clinical strains. n = 4 biological replicates for non-MDR INH-resistant TDR-TB 42 (red). n = 1 biological replicate for all other TDR-TB clinical strains. Data depicted as mean ± SEM. Source data are provided in the Source Data file.

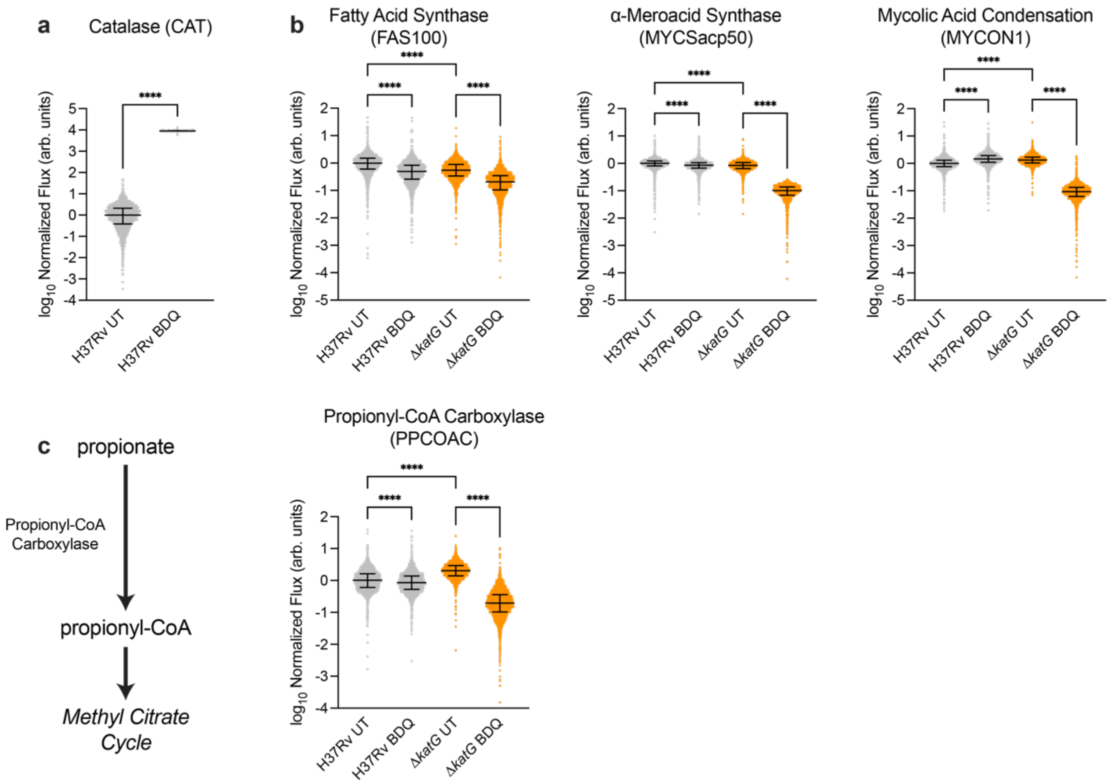

Supplementary Fig. 5. Bedaquiline grossly alters mycobacterial metabolism in Δ*katG* cells. a, Simulated catalase (CAT reaction) for BDQ-treated and untreated wild-type and Δ*katG* H37Rv cells from the iEK1011 Mtb genome-scale metabolic model. **b**, Simulated fatty acid synthase (FAS100), α-meroacid synthase (MYCSacp50), and mycolic acid condensation (MYCON1) activities for BDQ-treated and untreated wild-type and Δ*katG* H37Rv cells. BDQ synergistically represses mycolic acid synthesis in Δ*katG* cells. **c**, Simulated propionyl-CoA carboxylase (PPCOAC) activities for BDQ-treated and untreated wild-type and Δ*katG* H37Rv cells. BDQ synergistically represses propionate metabolism in Δ*katG* cells. n = 10,000 flux samples were collected for each metabolic simulation. Two-tailed Mann-Whitney or Kruskal-Wallis test was performed on metabolic modelling simulations with comparisons between untreated wild-type and Δ*katG* cells, BDQ-treated and untreated cells wild-type cells, and BDQ-treated and untreated Δ*katG* cells with Dunn's multiple comparisons test FDR correction, as indicated. ****: $p \leq 0.0001$. Data depicted as mean ± SEM. Source data are provided in the Source Data file.

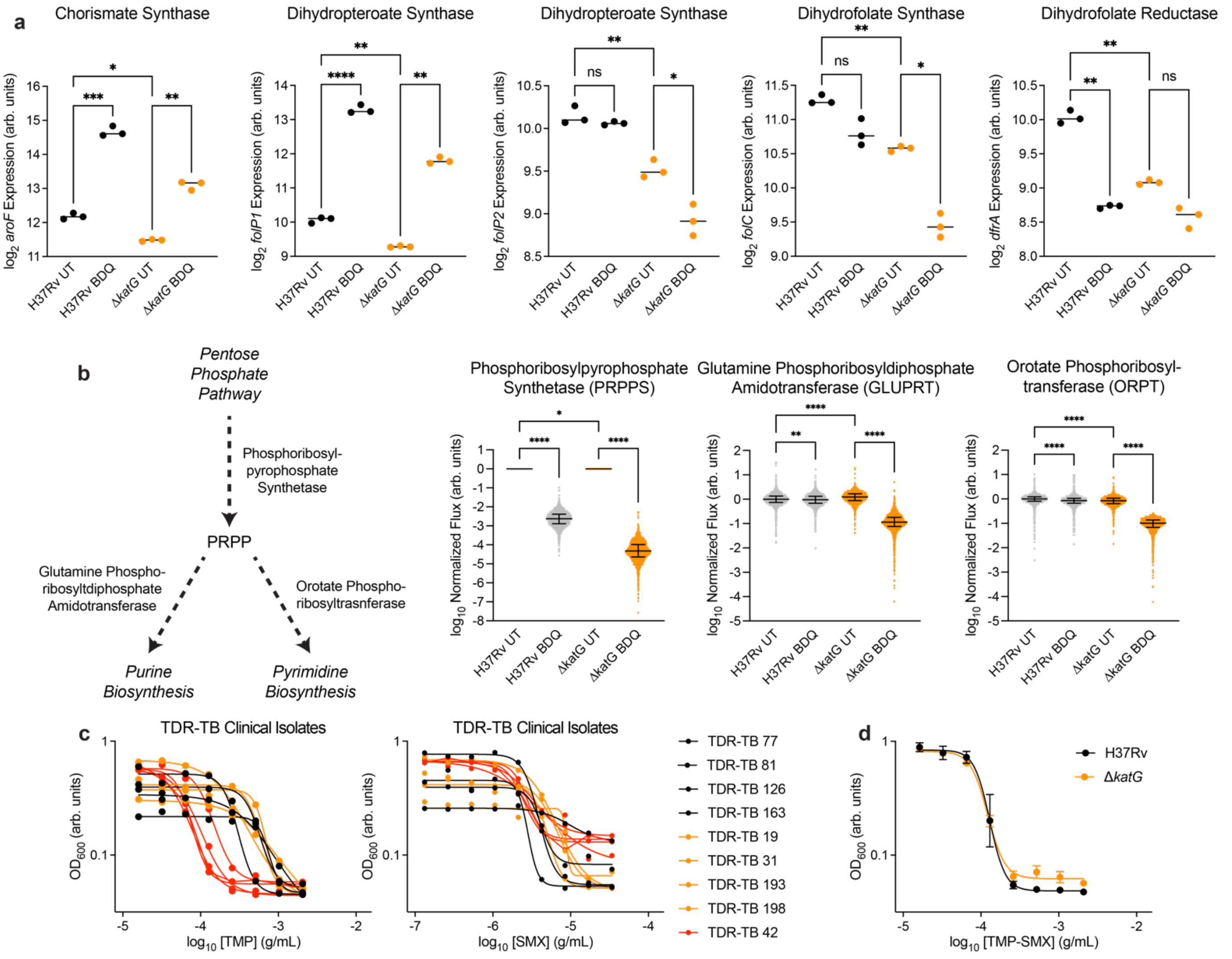

Supplementary Fig. 6. Catalase activity deficiency sensitizes Mtb to inhibition of folate biosynthesis. a, BDQ treatment increases *aroF* and *folP1* expression and decreases *folP2*, *folc*, and *dfrA* expression in wild-type and Δ*katG* H37Rv cells as measured by RNA sequencing. Expression data reported as smooth quantile normalized $\log_2$ sequencing counts. **b**, Simulated phosphoribosylpyrophosphate synthase (PRPPS reaction), glutamine phosphoribosyldiphosphate amidotransferase (GLUPRT), and orotate phosphoribosyltransferase (ORPT) activities for BDQ-treated and untreated wild-type and Δ*katG* H37Rv cells using the iEK1011 Mtb genome-scale metabolic model. n = 10,000 flux samples were collected for each metabolic simulation. **c**, 8-day TMP (left) and SMX (right) growth inhibition dose-response experiments for individual TDR-TB clinical strains (Fig. 5b and 5c). A non-MDR INH-resistant (TDR-TB 42) clinical strain is hypersensitive to both TMP and SMX relative to INH-susceptible (black) clinical strains. MDR clinical strains (orange) are not hypersensitive neither TMP nor SMX relative to INH-susceptible strains. n = 4 biological replicates for non-MDR INH-resistant TDR-TB 42 (red). n = 1 biological replicate for all other TDR-TB clinical strains. **d**, Δ*katG* cells are not sensitized to the combination of TMP and SMX relative to wild-type cells in 14-day growth inhibition dose-response experiments. n = 3 biological replicates. Brown-Forsythe and Welch ANOVA tests were performed on RNA expression data with comparisons between BDQ-treated and untreated cells wild-type and Δ*katG* cells or between untreated wild-type and Δ*katG* with Dunnett's T3 multiple comparisons test FDR correction, as indicated. *: $p \leq 0.05$, **: $p \leq 0.01$, ***: $p \leq 0.001$, ****: $p \leq 0.0001$. Data depicted as mean ± SEM. Source data are provided in the Source Data file.
