# Supplementary material for: Catalase activity deficiency sensitizes multidrug-resistant Mycobacterium tuberculosis to the ATP synthase inhibitor bedaquiline
Source: Nat Commun. 2024 Nov 13;15:9792. doi: 10.1038/s41467-024-53933-8 (PMC11561320; doi:10.1038/s41467-024-53933-8)
Supplement: Supplementary file 3 — Description of Additional Supplementary Files [file 41467_2024_53933_MOESM3_ESM.pdf]

Description of Additional Supplementary Files

Supplementary Data 1. Smooth quantile normalized RNA-sequencing gene expression profiles.

Supplementary Data 2. Hierarchically clustered genes from BDQ-treated and untreated wild-type and ΔkatG H37Rv cells.

Supplementary Data 3. iEK1011 metabolic modeling simulations.
